# Sex-specific single transcript level atlas of vasopressin and its receptor (AVPR1a) in the mouse brain

**DOI:** 10.7554/eLife.105355

**Published:** 2026-03-16

**Authors:** Anisa Azatovna Gumerova, Georgii Pevnev, Funda Korkmaz, Uliana Cheliadinova, Guzel Burganova, Darya Vasilyeva, Liam Cullen, Orly Barak, Farhath Sultana, Weibin Zhou, Steven Lee Sims, Emily Weiss, Victoria Laurencin, Tal Frolinger, Se-Min Kim, Ki A Goosens, Tony Yuen, Mone Zaidi, Vitaly Ryu

**Affiliations:** 1 Institute for Translational Medicine and Pharmacology, Icahn School of Medicine at Mount Sinai New York United States; Albert Einstein College of Medicine Bronx United States; SUNY Upstate Medical University Syracuse United States

**Keywords:** vasopressin, brain atlas, AVPR1a, Mouse

## Abstract

Vasopressin (AVP), a nonapeptide synthesized predominantly by magnocellular hypothalamic neurons, is conveyed to the posterior pituitary via the pituitary stalk, where AVP is secreted into the circulation. Known to regulate blood pressure and water homeostasis, it also modulates diverse social behaviors, such as pair bonding, social recognition, and cognition in mammals, including humans. Importantly, AVP modulates social behaviors in a sex-specific manner, perhaps due to sex differences in the distribution in the brain of AVP and its main receptor AVPR1a. There is a *corpus* of integrative studies for the expression of AVP and AVPR1a in various brain regions, and their functions in modulating central and peripheral actions. In order to purposefully address sexually dimorphic and novel roles of AVP on central and peripheral functions through its AVPR1a, we utilized RNAscope to map *Avp* and *Avpr1a* single transcript expression in the mouse brain. As the most comprehensive atlas of AVP and AVPR1a in the mouse brain, this compendium highlights the importance of newly identified AVP/AVPR1a neuronal nodes that may stimulate further functional studies.

## Introduction

Vasopressin (AVP), a nonapeptide synthesized primarily by magnocellular neurons of the paraventricular nucleus (PVH) and supraoptic nucleus (SO) of the hypothalamus, is conveyed along axons of magnocellular neurons via the pituitary stalk to the posterior pituitary, where AVP is released into circulation to exert hormonal functions (Brownstein et al., 1980; Young and Gainer, 2003). Involved in the regulation of blood pressure and water balance (Silva et al., 1969; Stockand et al., 2022), AVP also regulates diverse social behaviors, such as pair bonding, social recognition, and cognition in all mammals, including humans (Lukas and Neumann, 2013; Lukas et al., 2013; Veenema and Neumann, 2008; Meyer-Lindenberg et al., 2011; Albers, 2015). It should be noted that similar to OXT, AVP is evolutionarily conserved across invertebrates and vertebrate taxa, differing from OXT by just two amino acids. Importantly, AVP modulates social behaviors in a sex-specific manner, perhaps due to sex differences in AVP and its receptor expression in the brain (Winslow et al., 1993; Insel and Hulihan, 1995; Dantzer et al., 1987; Rigney et al., 2023; Landgraf et al., 2003; Veenema et al., 2012).

AVP receptors are G protein-coupled receptors consisting of two major subtypes, the V1 receptor (AVPR1) and V2 receptor (AVPR2) (Dumais and Veenema, 2016). AVPR1 has two subtypes, namely AVPR1a and AVPR1b, that mediate the effects of AVP on social behaviors. Avpr1a will be the overarching focus of this study given its abundance and ubiquity in brain regions (Albers, 2015). In fact, differences in AVPR1a genetic variability and expression patterns determine specific social phenotypes (French et al., 2016; Phelps et al., 2017; Lim et al., 2004; Fink et al., 2007; Ren et al., 2014). There is evidence that AVPR1a is involved in maternal care, pair bonding, behavioral aggression, anxiety, social recognition and social play (Lukas et al., 2013; Albers, 2015; Lim et al., 2004; Bielsky et al., 2004; Neumann and Landgraf, 2012; Lago et al., 2021; Thompson et al., 2006). The sex-dependent distribution of Avpr1a across the brain in different species provides a proxy for the distribution of AVP binding and, therefore, provides further evidence for central AVP neural nodes of physiological relevance.

Blocking AVPR1a inhibits social recognition in the rat, while AVPR1a knockout mice fail to display social recognition (Veenema et al., 2012; Bielsky et al., 2004; Bielsky et al., 2005). These effects of AVP in enhancing social recognition are mediated via AVPR1a in the lateral septum (Veenema et al., 2012; Bielsky et al., 2005). Sex differences in Avpr1a binding densities have been described in several brain sites of Wistar rats. Namely, males display higher AVPR1a binding densities in the following forebrain areas: somatosensory and piriform cortex, medial posterior bed nucleus of the stria terminalis (BNST), nucleus of the lateral olfactory tract (LOT), anteroventral thalamic nucleus (VA), tuberal lateral hypothalamus (LH), stigmoid hypothalamus (Stg), and dentate gyrus (DG) (Dumais and Veenema, 2016). In male prairie voles, central AVP infusion facilitates selective aggression associated with pair bond formation and partner preference, and the Avpr1a antagonist 1-(β-mercapto-β*,* β-cyclopentamethylene propionic acid) does not seem to inhibit aggression (Winslow et al., 1993). In contrast, central AVP infusion induces partner preference in female prairie voles (Insel and Hulihan, 1995). Comparative analysis of AVPR1a distribution has revealed higher densities of AVPR1a binding in the ventral pallidum, amygdala, and thalamus of prairie voles than that of meadow or montane voles (Insel et al., 1994; Wang et al., 1997). It has also been reported that AVPR1a antagonism specifically in the ventral pallidum prevents mating-induced partner preference in male prairie voles (Lim and Young, 2004).

Although osmotically stimulated AVP release with short latency and duration from hypothalamic PVH and SO magnocellular axon terminals occurs in the pituitary (Summy‐Long and Kadekaro, 2001; Stricker et al., 2002), it is also released locally from somata and dendrites in the SO with a longer delay responding to osmotic challenge (Ludwig et al., 1994; Ludwig and Leng, 1997). Such local release is likely to facilitate autocrine and/or paracrine regulation of SO magnocellular neuronal activity and inhibit further systemic AVP output (Ludwig and Leng, 1997; Wang et al., 1982). Of note, somatodendritic AVP release in response to direct hypertonic stimulation is attenuated by V1/V2 receptor antagonism, implying that AVP may facilitate its own release by acting on autoreceptors within magnocellular neurons of the SO (Wotjak et al., 1994). The importance of autofacilitation to local AVP release may lie in fine-tuned regulation of AVP actions toward physiological demands. Alternatively, AVP could putatively diffuse over longer distances to bind to adjacent receptors. The relative contribution of AVP autoreceptor subtypes, including AVPR1a, to this phenomenon awaits further clarification.

Current studies implicate posterior pituitary hormones, traditionally thought of as master regulators of a single physiological target, in the control of multiple bodily systems, either directly or via their receptors in the brain (Neumann and Landgraf, 2012; Zaidi et al., 2018; Carter, 2014; Koshimizu et al., 2012; Jurek and Neumann, 2018). Non-traditional actions of AVP include its ability to affect skeletal homeostasis, wherein it negatively regulates osteoblasts and stimulates osteoclasts. This explains the bone loss that accompanies hyponatremia in patients with elevated AVP levels (Tamma et al., 2013). Furthermore, we have shown that AVP (via AVPR1a) and oxytocin (via OXTR) have opposing skeletal actions—effects that may relate to the pathogenesis of bone loss in chronic hyponatremia, and pregnancy and lactation, respectively (Tamma et al., 2013; Sun et al., 2019; Sun et al., 2016; Tamma et al., 2009).

Detecting specific AVPR1a and AVPR1b in the brain has had limitations for a long time due to the availability of only nonselective radioligands, such as tritium labeled (^3^H) AVP ligands, which bind to both receptors (Phillips et al., 1990). Although there is a large body of integrative studies for the expression of AVP and AVPR1a in various brain regions, and their functions in regulating central and peripheral actions, there remains the need for detailed, sex-specific mapping of the AVP/AVPR1a neuronal nodes in the brain. We utilized RNAscope—a technology that detects single RNA transcripts—to create a comprehensive atlas of AVP and AVPR1a in the mouse brain. It may seem somewhat remarkable that newly discovered brain areas for receptors of such evolutionarily conserved and well-studied hormones AVP and OXT are currently emerging, with inferences to novel functions. We believe that this atlas of AVP and its AVPR1a in concrete brain sites at a single transcript level should provide a resource to neuroscientists to deepen our understanding of classical and novel central and peripheral functions of AVP by interrogating AVPR1a site-specifically.

## Results

AVP receptors are G protein-coupled receptors consisting of two major subtypes, AVPR1 and AVPR2 (Dumais and Veenema, 2016). AVPR1 is further divided into AVPR1a and AVPR1b receptor subtypes that mediate the effects of AVP in the brain on social behaviors. In this study, we have provided not only a distribution mapping of AVP and AVPR1a in the brain, but also assessed sex differences in their expression by RNAscope. Allowing the detection of single transcripts, RNAscope uses ~20 pairs of transcript-specific double *Z*-probes to hybridize 10-µm-thick whole brain sections. Preamplifiers first hybridize to the ~28 bp binding site formed by each double *Z*-probe; amplifiers then bind to the multiple binding sites on each preamplifier; and finally, labeled probes containing a chromogenic enzyme bind to multiple sites of each amplifier.

RNAscope data were quantified on every tenth section of the whole brains from coded three female and three male mice. For simplicity and clarity in the graphs, a scatter plot has been shown for three nuclei, subnuclei, and regions with the highest AVP and AVPR1a transcript densities, as well as their absolute transcript counts. Each section was viewed and analyzed using CaseViewer 2.4 (3DHISTECH, Budapest, Hungary) and QuPath v.0.2.3 (University of Edinburgh, UK). The *Atlas for the Mouse Brain in Stereotaxic Coordinates* (Paxinos and Franklin, 2007) was used to identify every nucleus, subnucleus, or region, which was followed by manual counting of *Avp* and *Avpr1a* transcripts by two independent observers (VR and AG) in every tenth section using the tag feature. Receptor density was calculated by dividing the absolute receptor number by the total area (µm^2^, ImageJ) of every nucleus, subnucleus, or region. The highest *Avp* and *Avpr1a* values in the brain regions are presented as means ± SE and compared with those of the opposite sex. Photomicrographs were prepared using Photoshop CS5 (Adobe Systems) only to adjust brightness, contrast, and sharpness, to remove artifacts (i.e. obscuring bubbles) and to make composite plates.

**Figure 1.**
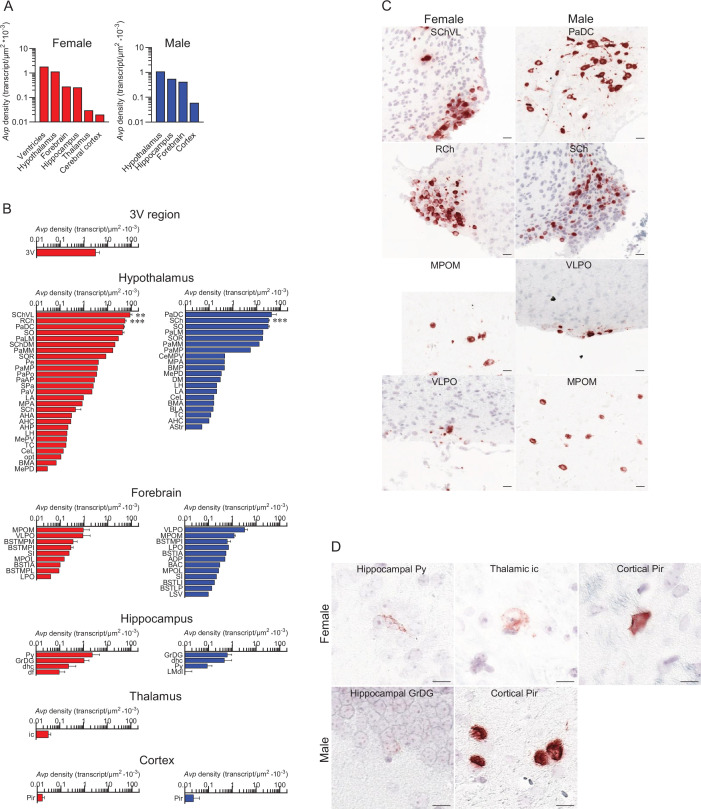
Sex-specific *Avp* transcript density in the brain. (**A**) *Avp* transcript density in main brain regions detected by RNAscope. Female 3V region and hypothalamus and male hypothalamus had the highest *Avp* transcript density. (**B**) *Avp* transcript density in nuclei, subnuclei, and regions of the hypothalamus and forebrain. (**C**) Representative micrographs of some of the hypothalamic and forebrain regions with highest *Avp* expression. Scale bar: 20 µm. (**D**) Novel *Avp* transcripts found in nuclei, subnuclei, and regions of the hippocampus, thalamus, and cerebral cortex. Scale bar: 10 µm. Note that *Avp* transcripts were found only in the female 3V ependymal layer and thalamus. N=3 mice *per* sex, values are shown as means ± SE . ***p*<*0.001 and **p*<*0.01, unpaired Student’s *t* test and two-way ANOVA.

RNAscope revealed *Avp* expression in the hypothalamus, forebrain, hippocampus, and cortex of both female and male mice (Figure 1A); however, *Avp* expression in the 3rd ventricular region and thalamus was found only in the female mouse (Figure 1B). Whereas the numbers of *Avp*-expressing cells were greater in females compared with males in the hypothalamus (607 vs 471), 3rd ventricular region (7 vs 0) and thalamus (2 vs 0), those numbers were lower in the hippocampus (58 vs 118), forebrain (50 vs 77) and cortex (5 vs 6) (see Appendix for Glossary and Figure 1—figure supplement 1 for raw count graphs).

**Figure 1—figure supplement 1.**
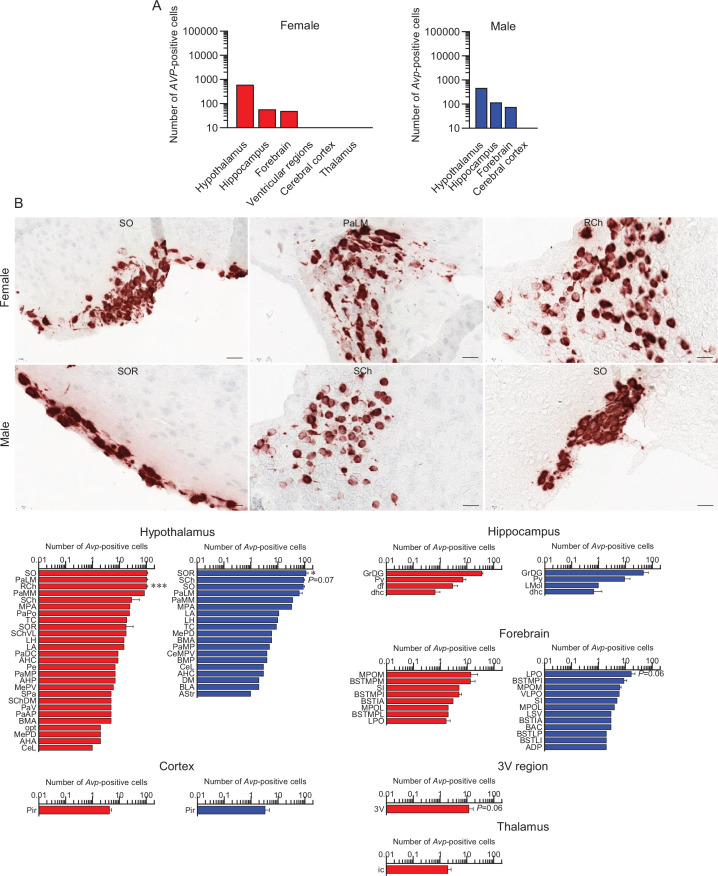
Sex-specific *Avp*-positive cells in the brain. (**A**) Total numbers of *Avp*-positive cells in main brain regions detected by RNAscope. (**B**) Total numbers of *Avp*-positive cells in nuclei, subnuclei, and regions of the hypothalamus, hippocampus, forebrain, and cortex. Note that *Avp*-positive cells were found only in the female 3V ependymal layer and thalamus. Also shown are representative RNAscope micrographs of hypothalamic regions with highest *Avp* expression; i.e*.*, supraoptic nucleus (SO), paraventricular hypothalamic nucleus, lateral magnocellular part (PaLM), retrochiasmatic area (RCh), supraoptic nucleus, retrochiasmatic part (SOR), and suprachiasmatic nucleus (SCh). Scale bar: 20 µm. N=3 mice *per* sex, values are shown as means ± SE . ***p*<*0.001 and *p*<*0.05, unpaired Student’s *t* test and two-way ANOVA.

The highest *Avp* transcript densities were detected in the following brain nuclei, subnuclei, and regions of female and male mice, respectively: ventricular region—3V only for females, hypothalamus—SChVL and PaDC, forebrain—MPOM and VLPO, hippocampus—Py and GrDG, thalamus—ic only for females, and cerebral cortex—Pir for both (Figure 1B). Representative micrographs of some of the hypothalamic and forebrain regions with highest *Avp* expression are shown in Figure 1C.

**Figure 2.**
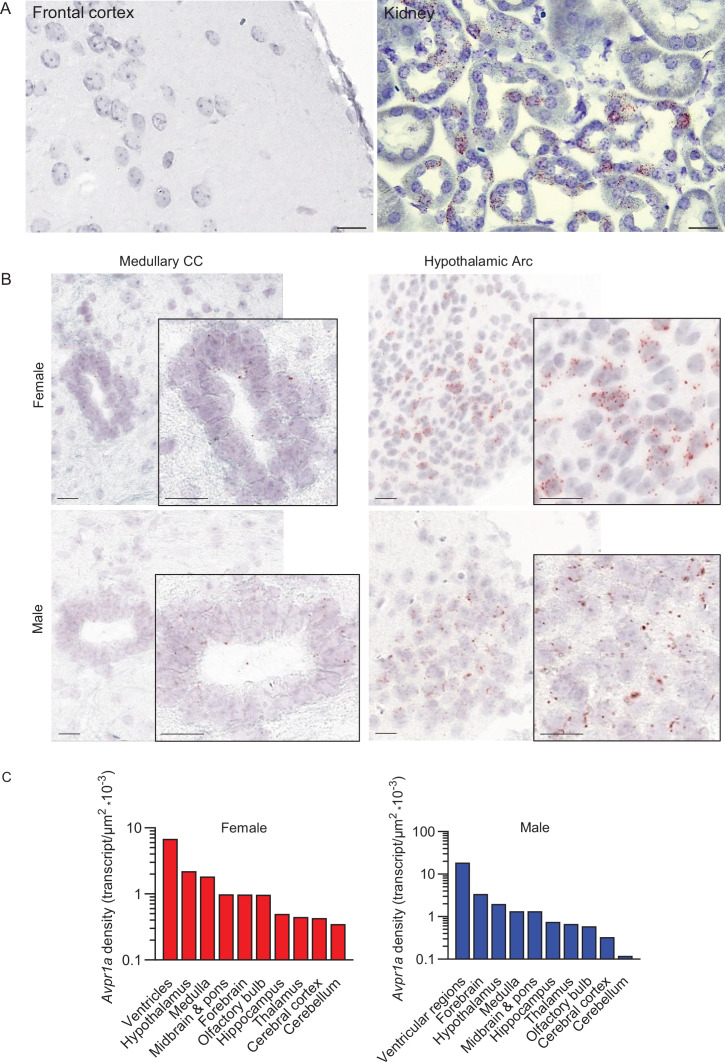
Sex-specific *Avpr1a* transcript density in main brain divisions. (**A**) Probe specificity was established by a positive signal in renal tubules of the kidney (positive control) with an absent signal in the frontal association cortex (FrA) (negative control). Sale bar: 20 µm. (**B**) Representative micrographs of medullary central canal (CC) and hypothalamic arcuate nucleus (Arc). (**C**) *Avpr1a* transcript density in main brain regions detected by RNAscope. N=3 mice *per* sex. Scale bar: 25 µm (controls).

We found that *Avpr1a* transcript expression in several brain nuclei, e.g., the MS of the forebrain, medullary 12N, IRt, LRt, MdV (Figure 2—figure supplement 1B), displayed individual patterns of expression compared with more ubiquitous and even expression noted in most of the other brain areas, suggesting brain-site-specific functional diversity and context-selective regulation of *Avpr1a*-mediated signaling. We report the expression of the *Avpr1a* in 398 and 375 brain nuclei, subnuclei, and regions of the female and male mice, respectively. *Avpr1a* transcripts were detected bilaterally, with no apparent ipsilateral dominance. Probe specificity was established by detecting a positive signal in renal tubules (positive control) with an absent signal in the FrA of the frontal cortex (negative control) (Figure 2A). Representative micrographs of the sex-specific medullary CC and hypothalamic Arc with highest *Avpr1a* expression are shown in Figure 2B.

**Figure 2—figure supplement 1.**
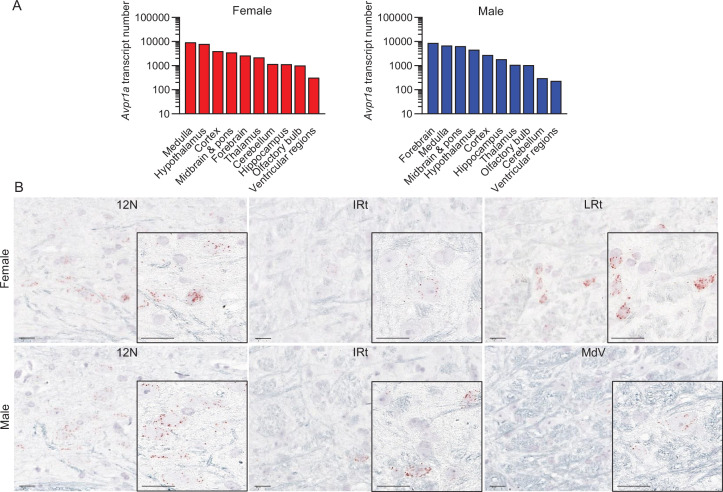
Sex-specific *Avpr1a* transcript numbers in main brain divisions. (**A**) Total *Avpr1a* transcript numbers in main brain regions detected by RNAscope. (**B**) Representative RNAscope micrographs of medullary regions with highest *Avpr1a* expression; i.e., hypoglossal nucleus (12N), intermediate reticular nucleus (IRt), lateral reticular nucleus (LRt), and medullary reticular nucleus, ventral part (MdV). N=3 mice *per* sex. N=3 mice *per* sex. Scale bar: 20 µm.

**Figure 3.**
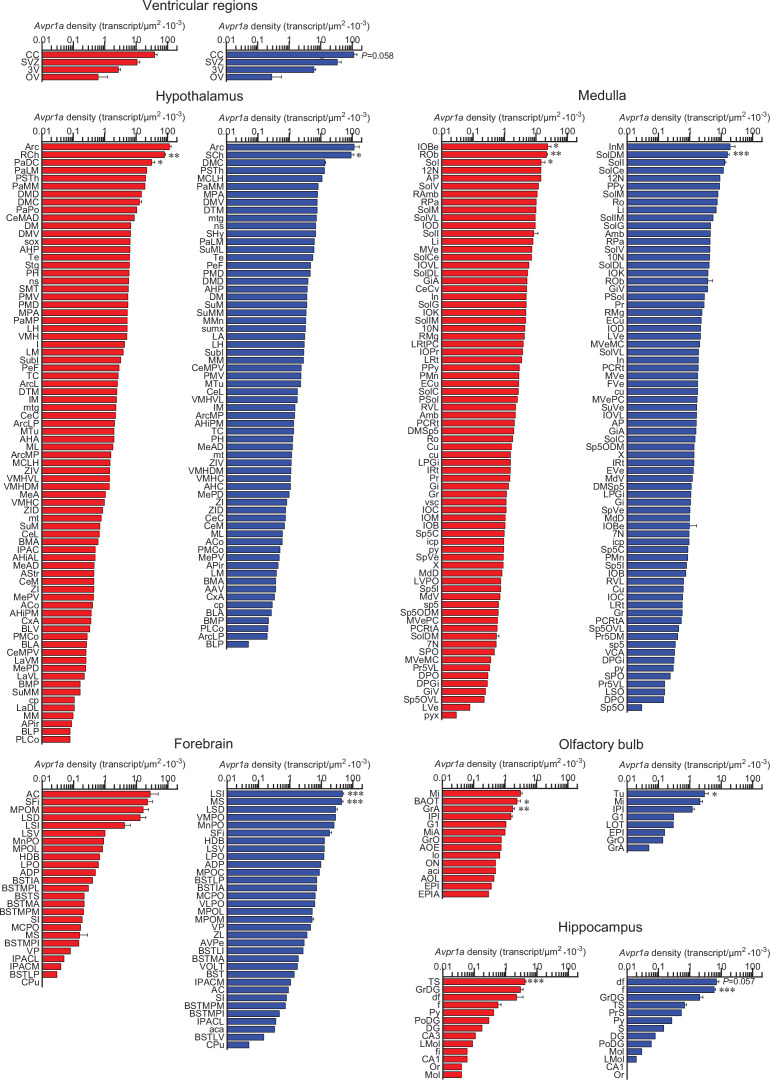
Sex-specific *Avpr1a* transcript density in the brain. *Avpr1a* transcript density in nuclei, subnuclei, and regions of the ventricular regions, hypothalamus, medulla, forebrain, olfactory bulb, and hippocampus. N=3 mice *per* sex, values are shown as means ± SE. ***p*<*0.001, **p*<*0.01, and *p*<*0.05, unpaired Student’s *t* test and two-way ANOVA.

**Figure 4.**
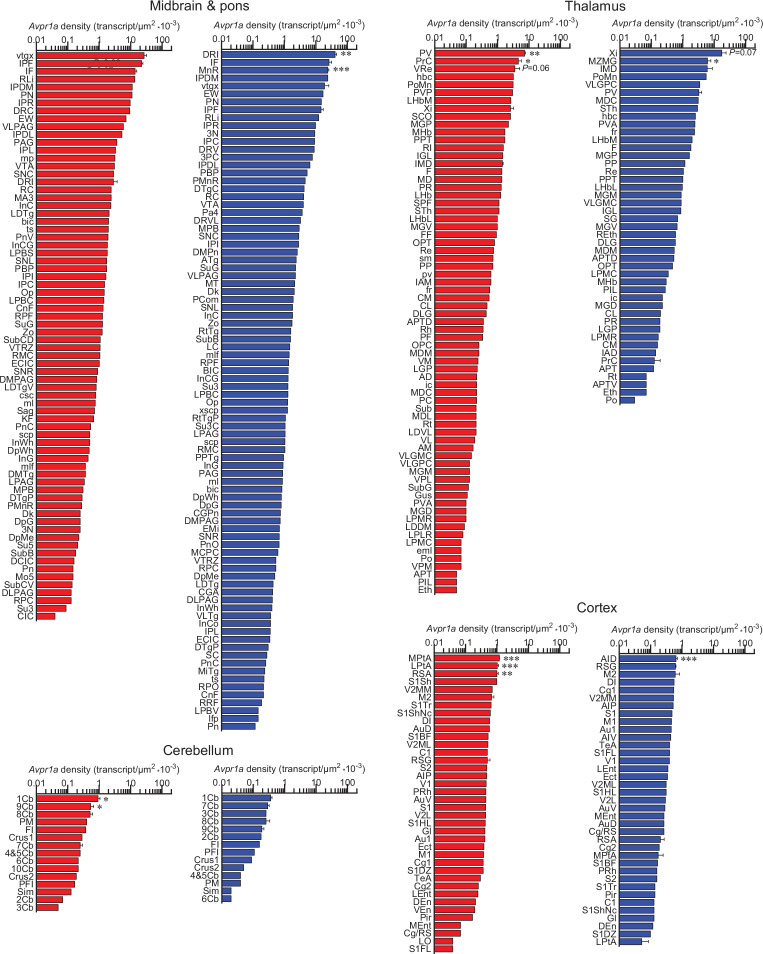
Sex-specific *Avpr1a* transcript density in the brain. *Avpr1a* transcript density in nuclei, subnuclei, and regions of the midbrain and pons, thalamus, cortex, and cerebellum. N=3 mice *per* sex, values are shown as means ± SE. ***p*<*0.001, **p*<*0.01, and *p*<*0.05, unpaired Student’s *t* test and two-way ANOVA.

In the female, total *Avpr1a* transcript numbers were the highest in the medulla followed in descending order by the hypothalamus, cortex, midbrain and pons, forebrain, thalamus, cerebellum, hippocampus, olfactory bulb, and ventricular regions (Figure 2—figure supplement 1A). In the male, *Avpr1a* transcripts were the highest in the forebrain, followed by the medulla, midbrain and pons, hypothalamus, cortex, hippocampus, thalamus, olfactory bulb, cerebellum, and ventricular regions (Figure 2—figure supplement 1A). Detailed *Avpr1a* transcript counts are shown in Figures 3 and 4.

**Figure 3—figure supplement 1.**
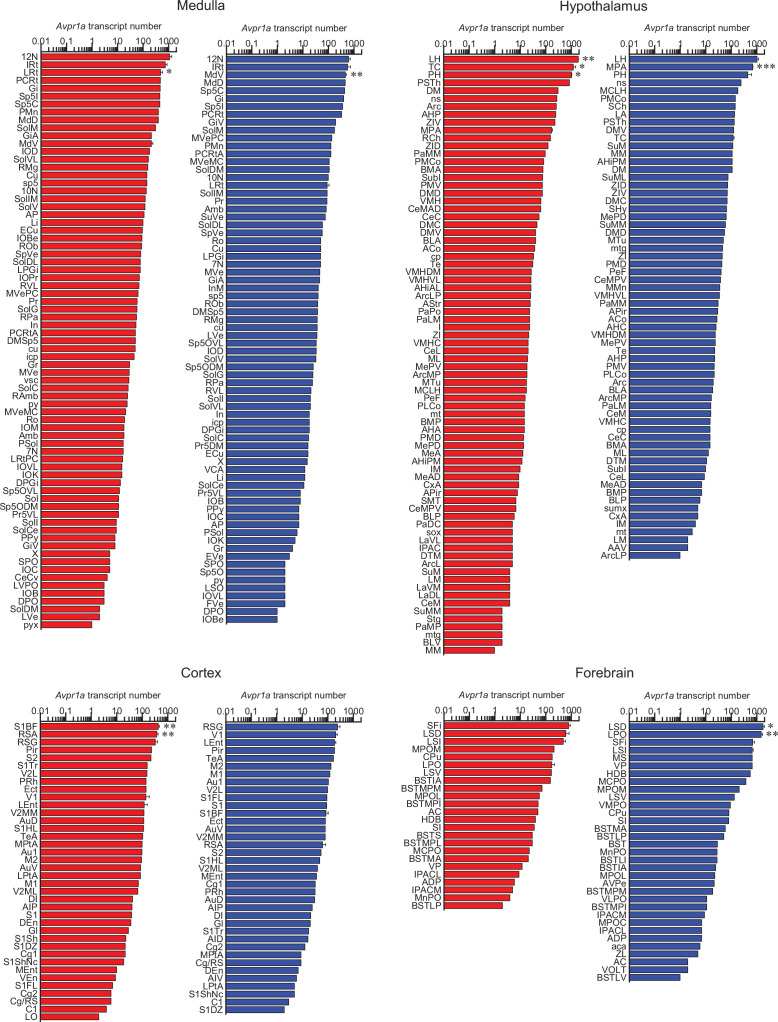
Sex-specific *Avpr1a* transcript numbers in the brain. *Avpr1a* transcript density in nuclei, subnuclei, and regions of the medulla, hypothalamus, cortex, and forebrain. N=3 mice *per* sex, values are shown as means ± SE. ***p*<*0.001, **p*<*0.01, and *p*<*0.05, unpaired Student’s *t* test and two-way ANOVA.

**Figure 4—figure supplement 1.**
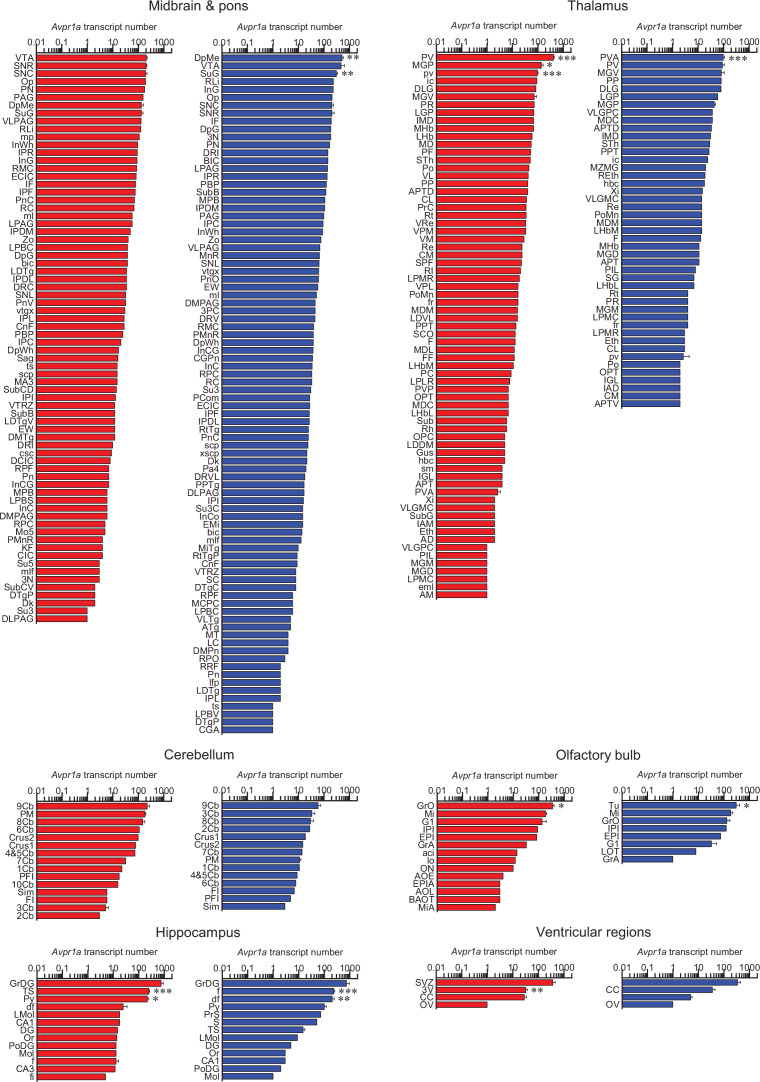
Sex-specific *Avpr1a* transcript numbers in the brain. *Avpr1a* transcript density in nuclei, subnuclei, and regions of the midbrain and pons, thalamus, cerebellum, olfactory bulb, hippocampus, and ventricular regions. N=3 mice *per* sex, values are shown as means ± SE. ***p*<*0.001, **p*<*0.01, and *p*<*0.05, unpaired Student’s *t* test and two-way ANOVA.

Using the RNAscope dataset, we further calculated *Avpr1a* density in all brain divisions (Figure 2C), nuclei, subnuclei, and regions (Figures 3 and 4). Highest *Avpr1a* densities in the female and male mice, respectively, were noted in nuclei, subnuclei, and regions as follows: ventricular regions—CC ependymal region for both with 2.92-fold greater in the male; hypothalamus—Arc for both; medulla—IOBe and InM; midbrain and pons—vtgx and DRI; forebrain—AC and LSI; olfactory bulb—Mi and Tu; hippocampus—TS and df; thalamus—PV and Xi; cortex—MPtA and AID; and cerebellum—1Cb for both with 2.58-fold greater in the female (Figures 3 and 4).

**Figure 5.**
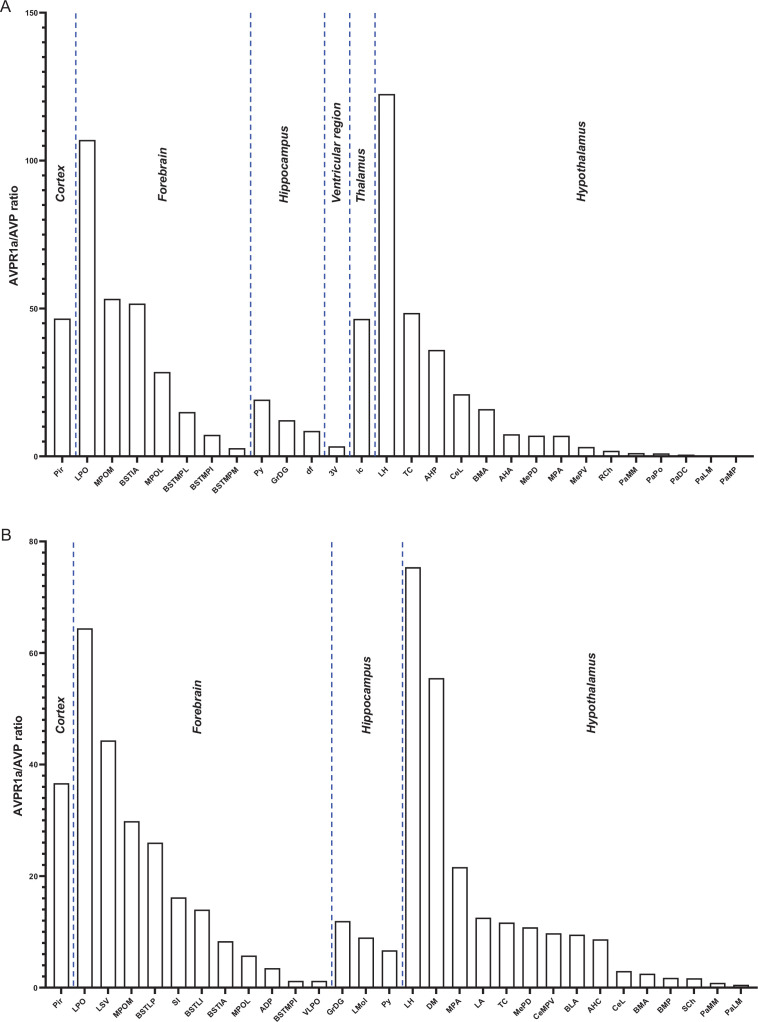
*Avp* and *Avpr1a* co-localization in the brain. We found that various nuclei and subnuclei exhibited *Avp* and *Avpr1a* co-localization in the brain of both sexes. (**A**) Female and (**B**) male *Avpr1a* to *Avp* ratios in the cortex, forebrain, hippocampus, 3rd ventricular region, thalamus, and hypothalamus.

**Figure 6.**
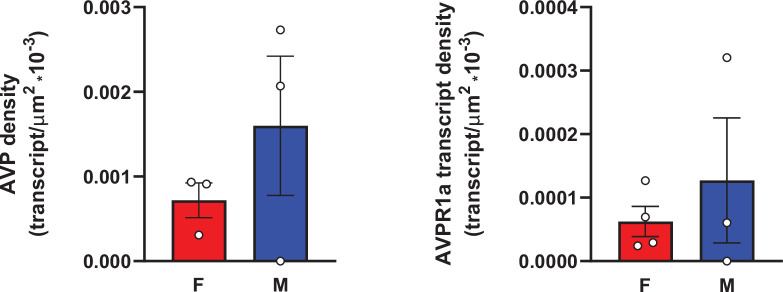
*Avp* and *Avpr1a* transcript densities in the pituitary gland. RNAscope revealed *Avp* and *Avpr1a* expression in the posterior pituitary lobe with *Avp* and *Avpr1a* transcript densities that were higher in male compared with female mice. N=3 mice *per* sex, values are shown as means ± SE. Unpaired Student’s *t* test.

In addition, RNAscope analysis revealed that various brain nuclei, subnuclei, and regions in both female and male mice co-localized *Avp* and *Avpr1a* transcripts. *Avpr1a* to *Avp* ratios within the same brain nucleus, subnucleus, and region in both sexes are demonstrated in Figure 5. Finally, RNAscope showed *Avp* and *Avpr1a* expression in the posterior pituitary lobe with *Avp* and *Avpr1a* transcript densities that were higher in male compared with female mice, however, without statistical significance (Figure 6).

## Discussion

Here, we attempted to integrate previous information on sex-specific AVP and its AVPR1a expression in the murine brain. AVPR1a is the most abundant and widespread receptor in the brain (Albers, 2015) that plays a dominant role in regulating behavior. In addition, we focused on paradigm-shifting non-traditional roles of central AVP signaling in light of newly discovered AVPR1a. We report AVP expression in 41 female and 13 male brain nuclei, subnuclei, and regions. Moreover, we identified abundant AVPR1a expression in 398 female and 375 male brain nuclei, subnuclei, and regions. Therefore, this report is the most exhaustive atlas of brain *Avp* and *Avpr1a* expression at the single transcript level.

It has been reported that AVP synthesis and AVP fiber projections are sexually dimorphic in specific brain sites (for review, see: Dumais and Veenema, 2016). To our knowledge, the first discovery of the sexually dimorphic nature of AVP in the rat brain was made by de Vries et al., 1981. That is, males displayed more AVP-immunoreactive fibers in the lateral septum and lateral habenular nucleus over females (de Vries et al., 1981). Surprisingly, sex differences in AVP fiber density in the LS and medial amygdala (MeA) originate from the BNST, given only lesions to the BNST, but not the PVH, result in decreased AVP fiber density in the LS (de Vries and Buijs, 1983; Caffé et al., 1989). In adult rats, AVP fiber density from the BNST and MeA is dependent on circulating gonadal hormones, as gonadectomy eliminates AVP expression and hormone replacement restores AVP fiber network (de Vries et al., 1984; Miller et al., 1992). Nonetheless, gonadal hormones appear to only partially explain sex differences in AVP expression because both females and males, exposed to a similar gonadal steroid hormone regime, still differ sexually (de Vries and al-Shamma, 1990; de Vries et al., 1994).

Magnocellular neurons of the PVN, SO, and SCh of the hypothalamus are the predominant source of AVP synthesis. Hypothalamic AVP synthesis in most rodent species is similar between males and females in the PVH and SO of mice (Joca et al., 2014; Steinman et al., 2015); PVH, SO, and SCh of voles (Wang, 1995; Wang et al., 1996); PVH, MPOA, LH, and AHA of Mongolian gerbils and Chinese striped hamsters (Wang et al., 2013) (for review, see Dumais and Veenema, 2016). In concordance with these reports, we also found no sex differences in *Avp* synthesis, as evidenced by similar numbers of *Avp*-expressing neurons in the PVH and SO of mice; however, we did note sex differences in *Avp* expression density in specific hypothalamic nuclei. Notably, *Avp* expression density was higher in several PVH (PaLM and PaMM) and suprachiasmatic (SChVL and RCh) subnuclei of female compared to male mice. No sex differences in SO*-Avp* expression density were found.

Furthermore, *Avpr1a* transcript density was highest in the arcuate nucleus (Arc) and retrochiasmatic subnucleus (RCh) in female compared with Arc and suprachiasmatic nucleus (SCh) of male mice. *Avpr1a* expression in the Arc has previously been reported (Ostrowski et al., 1992); however, its role was unclear until a recent report demonstrating a critical involvement of Arc-NPY in the regulation of fluid homeostasis and the induction of salt water-induced hypertension through AVP modulation in the SO (Zhang et al., 2022); the latter receives direct projections from the Arc (Pineda et al., 2016). In contrast, it is plausible, but by no means proven, that PVH- and/or SO-AVP may modulate Arc anorexigenic neurons to inhibit ingestive behavior, which has previously been shown with PVH oxytocinergic neurons (Maejima et al., 2014). Indeed, increasing evidence suggests that AVP reduces feeding in mammals (Meyer et al., 1989; Langhans et al., 1991).

It has been reported that in the rat, AVP is an important output of the SCh targeting AVP cells in other hypothalamic areas—its release into the CSF peaks in the early morning and declines later in a day (Kalsbeek et al., 2010). Specifically, SCh-AVP secreted during late sleep activates osmosensory afferents to AVP neurons in the SO and organum vasculosum of the lamina terminalis (Trudel and Bourque, 2010; Gizowski et al., 2016). Similar to rodents, studies in humans also determined that the main AVP projections from the SCh target the anteroventral hypothalamic area, sub-PVH, as well as ventral parts of the PVH and DMH—a remarkable evolutionary conservation of SCh innervation from rodent to human (Dai et al., 1998a; Dai et al., 1998b). The fact that the SCh is another brain nucleus with high AVP and AVPR1a expression density (greater in males vs females) accentuates an important role of SCh-AVP in circadian rhythmicity, notably impacting neuroendocrine day/night rhythms, feeding timing, period, precision, and synchronization of SCh neurons (Kalsbeek et al., 2010; Rohr et al., 2021; Yoshimura et al., 2021).

In the hindbrain, the highest *Avpr1a* transcript density was noted in the inferior olive, beta subnucleus (IOBe) of female mice, and intermedius nucleus (InM) of male mice. It has been reported that AVP fibers are apparent in the hindbrain, such as the parabrachial nucleus, locus coeruleus, and near inferior olive nuclei (Young et al., 1999). In this regard, *Avpr1a* mRNA expression has been noted in the inferior olive (Ostrowski et al., 1992). Given this nucleus has been implicated in various functions, including learning and timing of movements, it is possible that AVPR1a in the inferior olive may be activated by the paracrine release of AVP from distant nuclei, such as the SCh, to control motor learning and timing. Alternatively, AVPR1a in the inferior olive may respond to other ligands (e.g., OXT) found in nearby regions (Szczepanska-Sadowska et al., 2021). The role of AVPR1 in the InM of male mice is less clear, but because the InM sends monosynaptic projections to the NTS (Edwards et al., 2009) that is essential for blood pressure control by AVP and receiving information from the cardiovascular receptors (Zanutto et al., 2010), a possible coordinated control by hindbrain AVP of blood pressure and cardiovascular function.

Although the midbrain, pons, and forebrain displayed less abundant *Avpr1a* transcript density, they revealed further sex differences. In the midbrain and pons, the highest *Avpr1a* density was observed in the ventral tegmental decussation (vtgx) in females and dorsal raphe nucleus, interfascicular part (DRI), in males. AVP and OXT in the ventral tegmental area are known to regulate social interactions with rewarding properties. Indeed, humans, as inherently social beings, show a strong inclination to affiliate and share their emotions with each other (Baumeister and Leary, 1995; Wagner et al., 2015). Sex differences in ventral tegmental AVPR1a make biological sense, as social interaction of females, specifically, with pups and, generally, with counterparts throughout their lives, has rewarding properties fundamental to maternal behavior and survival. Modulation of AVPR1a in the dorsal raphe nucleus has also been linked to social and emotional behaviors (Dumais and Veenema, 2016; Rigney et al., 2020; Rood et al., 2013). Sexual dimorphism in AVP innervation of and AVPR1a expression in the DRI appears to imprint dimorphic social behaviors. That is, AVPR1a blockade in the lateral habenular nucleus (LHb) of males, but not females who have lesser AVP innervation of the LHb and dorsal raphe nucleus, results in reduced urine marking to unfamiliar males and ultrasonic vocalizations to unfamiliar, sexually receptive females, whereas AVPR1a blockade in the dorsal raphe nucleus of only males reduces urine marking to unfamiliar males (Rigney et al., 2020).

In the forebrain, both sexes displayed high *Avpr1a* transcript density in septal nuclei. The highest *Avpr1a* density was in the anterior commissural nucleus (AC) within the septal nuclei of females, and lateral (LSI) and medial (MS) septal nuclei of males. It is not surprising that *Avpr1a* transcript density was significantly greater in septal nuclei of males than females, given that in many rodent species males have more AVP-immunoreactive fibers in the lateral septum (de Vries et al., 1981). Notably, the effects of AVP on social recognition is mediated via AVPR1a in the lateral septum (Veenema et al., 2012; Bielsky et al., 2005). For example, AVPR1a blockade inhibits social recognition in the rat, while AVPR1a knockout mice fail to display social recognition (Veenema et al., 2012; Bielsky et al., 2004; Bielsky et al., 2005).

Despite *Avpr1a* transcript densities in other brain divisions being significantly lower, sexually dimorphic differences are worth mentioning here. In the olfactory bulb, females had the high *Avpr1a* density in the mitral cell layer (Mi), whereas males had high receptor expression in the olfactory tubercle (Tu). A population of AVP neurons in the olfactory bulb of the rat that plays a role in social recognition via AVPR1a has been reported (Tobin et al., 2010). Silencing the AVPR1a by siRNA impairs habituation/dishabituation to juvenile cues, but not to volatile odors (Tobin et al., 2010). Of note, AVP is a retrograde signal that filters activation of the Mi cells in the ewe, likely through presynaptic modulation of norepinephrine or acetylcholine. The secretion of both transmitters is stimulated by AVP in the olfactory bulb (Tobin et al., 2010; Lévy et al., 1995). The functional relevance of AVP signaling via AVPR1a activation in the Tu requires additional studies. There is, however, evidence in the rat that AVP via AVPR1a has, at least, an indirect impact on Tu function, as seen by a reduction in activation responding to a noxious odor of butyric acid, when the AVPR1a is blocked (Reed et al., 2013). The presence of AVPR1a in the hippocampus, thalamus, cortex, and ventricular regions is consistent with the reported effects of AVP on memory (de Wied, 1971), emotional and reward-motivated behavior (Zhang et al., 2006), blood pressure (Matsuguchi et al., 1982), blood flow, and CSF production (Faraci et al., 1988). Functional roles of many other nuclei shown here to express AVPR1a and not mentioned in this report are much less clear. The importance of revealing novel AVP-triggered functions by interrogating AVPR1a site-specifically will require further investigations.

Collectively, our results provide compelling evidence of distinct and novel AVP/AVPR1a neuronal nodes in the brain. While studies on central AVP signaling and its control of blood pressure, water balance, and diverse social behaviors in mammals occupy the vast majority of the literature (Silva et al., 1969; Stockand et al., 2022; Lukas and Neumann, 2013; Lukas et al., 2013; Veenema and Neumann, 2008; Meyer-Lindenberg et al., 2011; Albers, 2015), we expect that this comprehensive compendium of sex-specific AVP/AVPR1a expression in the brain will deepen our understanding of the functional and neuroanatomical basis underlying old and new paradigm-shifting functions of central AVP signaling. As appears to be the case for most brain areas, the original discovery of function tends to become dogma, thereby leading to an oversimplification of multiple functions of those brain areas as they interact in circuits. Finally, the approach of direct mapping of receptor expression in the brain and periphery provides the groundwork for greater discernment of new functional arrangements of ancient pituitary glycoprotein hormones and nonapeptides, such as AVP and OXT, and provides helpful pointers toward improving pharmacological interventions in disease.

## Methods

### Mice

Adult C57BL/6J mice (~3- to 4-month-old) were housed in a 12 hr:12 hr light:dark cycle at 22 ± 2°C with ad libitum access to water and regular chow. All procedures were approved by the Mount Sinai Institutional Animal Care and Use Committee and are in accordance with Public Health Service and United States Department of Agriculture guidelines. Ethical approval for all experimental procedures was obtained from the appropriate Institutional Review Board under protocol number PROTO202100038.

### RNAscope

For RNAscope, mice were anesthetized with isoflurane (2–3% in oxygen; Baxter Healthcare, Deerfield, IL, USA) and transcardially perfused with 0.9% heparinized saline followed by 10% Neutral Buffered Formalin (NBF). Brains were promptly extracted, post-fixed in 10% NBF for 24 hr, dehydrated, and paraffin-embedded. Coronal sections were cut at 5 μm, with every tenth section mounted onto ~60 slides with 3 sections on each slide. This method allows covering the entire brain and eliminates the likelihood of counting the same transcript twice. Sections were air-dried overnight at room temperature and stored at 4°C until required.

Detection of mouse *Avp* and *Avpr* (*Avpr1a*) was performed separately on paraffin sections using Advanced Cell Diagnostics (ACD) RNAscope 2.5 LS Reagent Kit (#322100) and two RNAscope 2.5 LS probes, namely Mm-AVP-O1 (#472268) and Mm-AVPR1a (#418068). The kidney and prefrontal cortex served as positive and negative controls for AVPR1a, respectively. As with AVP, magnocellular cells of the PVH and SON served as positive controls, while the brain from the AVP knockout mouse served as a negative control.

Slides were baked at 60°C for 1 hr, deparaffinized, incubated with hydrogen peroxide for 10 min at room temperature, pretreated with Target Retrieval Reagent (#322001) for 20 min at 100°C and then with Protease III for 30 min at 40°C. Probe hybridization and signal amplification were performed as per the manufacturer’s instructions for chromogenic assays.

Following the RNAscope assay, the slides were scanned at ×20 magnification, and the digital image analysis was successfully validated using the CaseViewer 2.4 software (3DHISTECH). The same software was employed to capture and prepare images for the figures in the article. Images of control tissues were taken using the microscope Leica DM 1000 LED. Detection of *Avp*- and *Avpr1a*-positive cells was also performed using the QuPath-0.2.3 (University of Edinburgh, UK) software. *The Atlas for the Mouse Brain in Stereotaxic Coordinates* (Paxinos and Franklin, 2007) was utilized to identify and manually map every nucleus, subnucleus, or region using the drawing features of the QuPath-0.2.3 software in every tenth brain section. This was followed by exhaustive counting of *Avp* and *Avpr1a* transcripts using a tag feature. *Avp* and *Avpr1a* transcript density was calculated by dividing the absolute numbers by the total area (µm^2^, ImageJ) of every nucleus, subnucleus, or region. Photomicrographs were prepared using Photoshop CS5 (Adobe Systems) only to adjust brightness, contrast, and sharpness, to remove artifacts (e.g*.,* obscuring bubbles) and to make composite plates.

### Quantitation, validation, and statistical analysis

Data were analyzed by Student’s *t*-test and two-way analysis of variance (ANOVA) followed by Tukey’s multiple comparisons tests using GraphPad Prism 10.2.2 version (La Jolla, CA, USA). Significance was set at p<0.05. p-values are shown.

## Data Availability

The authors uploaded the dataset in Dryad to maintain high standards of research reproducibility. The following dataset was generated: GumerovaAA PevnevG KorkmazF CheliadinovaU BurganovaG VasilyevaD CullenL BarakO SultanaF ZhouW SimsSL WeissE LaurencinV FrolingerT KimS GoosensKA YuenT ZaidiM RyuV 2026Sex-specific Single Transcript Level Atlas of Vasopressin and its Receptor (AVPR1a) in the Mouse BrainDryad Digital Repository10.5061/dryad.np5hqc08h41837842

## Additional files

MDAR checklist

## Appendix 1

**Appendix 1—table 1.** Glossary of the brain nuclei, sub–nuclei and regions.

Olfactory bulb	
aci	anterior commissure, intrabulbar part
AOE	anterior olfactory nucleus, external part
AOL	anterior olfactory nucleus, lateral part
BAOT	bed nucleus of the accessory olfactory tract
EPI	external plexiform layer of the olfactory bulb
EPIA	external plexiform layer of the accessory olfactory bulb
G1	glomerular layer of the olfactory bulb
GrA	granule cell layer of the accessory olfactory bulb
GrO	granular cell layer of the olfactory bulb
IPI	interpeduncular nucleus, intermediate subnucleus
lo	lateral olfactory tract
LOT	nucleus of the lateral olfactory tract
Mi	mitral cell layer of the olfactory bulb
MiA	mitral cell layer of the accessory olfactory bulb
ON	olfactory nerve layer
Tu	olfactory tubercle
Cerebral cortex	
AID	agranular insular cortex, dorsal part
AIP	agranular insular cortex, posterior part
AIV	agranular insular cortex, ventral part
Au1	primary auditory cortex
AuD	secondary auditory cortex, dorsal area
AuV	secondary auditory cortex, ventral area
Cg/RS	cingular/retrosplenial cortex
Cg1	cingulate cortex, area 1
Cg2	cingulate cortex, area 2
C1	C1 adrenaline cells
DEn	dorsal endopiriform nucleus
DI	dysgranular insular cortex
Ect	ectorhinal cortex
GI	granular insular cortex
LEnt	lateral entorhinal cortex
LO	lateral orbital cortex
LPtA	lateral parietal association cortex
M1	primary motor cortex
M2	secondary motor cortex
MEnt	medial entorhinal cortex
MPtA	medial parietal association cortex
Pir	piriform cortex
PRh	perirhinal cortex
RSA	retrosplenial agranular cortex
RSG	retrosplenial granular cortex
S1	primary somatosensory cortex
S1BF	primary somatosensory cortex, barrel field
S1DZ	primary somatosensory cortex, dysgranular region
S1FL	primary somatosensory cortex, forelimb region
S1HL	primary somatosensory cortex, hindlimb region
S1Sh	primary somatosensory cortex, shoulder region
S1ShNc	primary somatosensory cortex, shoulder/neck region
S1Tr	primary somatosensory cortex, trunk region
S2	secondary somatosensory cortex
TeA	temporal association cortex
V1	primary visual cortex
V2L	secondary visual cortex, lateral area
V2ML	secondary visual cortex, mediolateral area
V2MM	secondary visual cortex, mediomedial area
VEn	ventral endopiriform nucleus
Forebrain	
AC	anterior commissural nucleus
aca	anterior commissure, anterior part
ADP	anterodorsal preoptic nucleus
AVPe	anteroventral periventricular nucleus
BAC	bed nucleus of the anterior commissure
BST	bed nucleus of the stria terminalis
BSTIA	bed nucleus of the stria terminalis, intraamygdaloid division
BSTLI	bed nucleus of the stria terminalis, lateral division, intermediate part
BSTLP	bed nucleus of the stria terminalis, lateral division, posterior part
BSTLV	bed nucleus of the stria terminalis, lateral division, ventral part
BSTMA	bed nucleus of the stria terminalis, medial division, anterior part
BSTMPI	bed nucleus of the stria terminalis, medial division, posterointermediate part
BSTMPL	bed nucleus of the stria terminalis, medial division, posterolateral part
BSTMPM	bed nucleus of the stria terminalis, medial division, posteromedial part
BSTS	bed nucleus of stria terminalis, supracapsular part
CPu	caudate putamen (striatum)
HDB	nucleus of the horizontal limb of the diagonal band
IPACL	interstitial nucleus of the posterior limb of the anterior commissure, lateral part
IPACM	interstitial nucleus of the posterior limb of the anterior commissure, medial part
LPO	lateral preoptic area
LSD	lateral septal nucleus, dorsal part
LSI	lateral septal nucleus, intermediate part
LSV	lateral septal nucleus, ventral part
MCPO	magnocellular preoptic nucleus
MnPO	median preoptic nucleus
MPOC	medial preoptic nucleus, central part
MPOL	medial preoptic nucleus, lateral part
MPOM	medial preoptic nucleus, medial part
MS	medial septal nucleus
SFi	septofimbrial nucleus
SI	substantia innominata
VLPO	ventrolateral preoptic nucleus
VMPO	ventromedial preoptic nucleus
VOLT	vascular organ of the lamina terminalis
VP	ventral pallidum
ZL	zona limitans
Hippocampus	
CA1	field ca1 of hippocampus
CA3	field CA3 of hippocampus
df	dorsal fornix
DG	dentate gyrus
dhc	dorsal hippocampal commissure
f	fornix
fi	fimbria of the hippocampus
GrDG	granular layer of the dentate gyrus
LMol	lacunosum moleculare layer of the hippocampus
Mol	molecular layer of the dentate gyrus
Or	oriens layer of the hippocampus
PoDG	polymorph layer of the dentate gyrus
PrS	presubiculum
Py	pyramidal tract
S	subiculum
TS	triangular septal nucleus
Thalamus	
AD	anterodorsal thalamic nucleus
AM	anteromedial thalamic nucleus
APT	anterior pretectal nucleus
APTD	anterior pretectal nucleus, dorsal part
APTV	anterior pretectal nucleus, ventral part
CL	centrolateral thalamic nucleus
CM	central medial thalamic nucleus
DLG	dorsal lateral geniculate nucleus
eml	external medullary lamina
Eth	ethmoid thalamic nucleus
F	nucleus of the fields of Forel
FF	fields of Forel
fr	fasciculus retroflexus
Gus	gustatory thalamic nucleus
hbc	habenular commissure
IAD	interanterodorsal thalamic nucleus
IAM	interanteromedial thalamic nucleus
ic	internal capsule
IGL	intergeniculate leaf
IMD	intermediodorsal thalamic nucleus
LDDM	laterodorsal thalamic nucleus, dorsomedial part
LDVL	laterodorsal thalamic nucleus, ventrolateral part
LGP	lateral globus pallidus
LHb	lateral habenular nucleus
LHbL	lateral habenular nucleus, lateral part
LHbM	lateral habenular nucleus, medial part
LPLR	lateral posterior thalamic nucleus, laterorostral part
LPMC	lateral posterior thalamic nucleus, mediocaudal part
LPMR	lateral posterior thalamic nucleus, mediorostral part
MD	mediodorsal thalamic nucleus
MDC	mediodorsal thalamic nucleus, central part
MDL	mediodorsal thalamic nucleus, lateral part
MDM	mediodorsal thalamic nucleus, medial part
MGD	medial geniculate nucleus, dorsal part
MGM	medial geniculate nucleus, medial part
MGP	medial globus pallidus (entopeduncular nucleus)
MGV	medial geniculate nucleus, ventral part
MHb	medial habenular nucleus
MZMG	marginal zone of the medial geniculate
OPC	oval paracentral thalamic nucleus
OPT	olivary pretectal nucleus
PC	paracentral thalamic nucleus
PF	parafascicular thalamic nucleus
PIL	posterior intralaminar thalamic nucleus
Po	posterior thalamic nuclear group
PoMn	posteromedian thalamic nucleus
PP	peripeduncular nucleus
PPT	posterior pretectal nucleus
PR	prerubral field
PrC	precommissural nucleus
pv	periventricular fiber system
PV	paraventricular thalamic nucleus
PVA	paraventricular thalamic nucleus, anterior part
PVP	paraventricular thalamic nucleus, posterior part
Re	reuniens thalamic nucleus
REth	retroethmoid nucleus
Rh	rhomboid thalamic nucleus
RI	rostral interstitial nucleus of medial longitudinal fasciculus
Rt	reticular thalamic nucleus
SCO	subcommissural organ
SG	suprageniculate thalamic nucleus
sm	stria medullaris of the thalamus
SPF	subparafascicular thalamic nucleus
STh	subthalamic nucleus
Sub	submedius thalamic nucleus
SubG	subgeniculate nucleus
VL	ventrolateral thalamic nucleus
VLGMC	ventral lateral geniculate nucleus, magnocellular part
VLGPC	ventral lateral geniculate nucleus, parvicellular part
VM	ventromedial thalamic nucleus
VPL	ventral posterolateral thalamic nucleus
VPM	ventral posteromedial thalamic nucleus
VRe	ventral reuniens thalamic nucleus
Xi	xiphoid thalamic nucleus
Hypothalamus	
AAV	anterior amygdaloid area, ventral part
ACo	anterior cortical amygdaloid nucleus
AHA	anterior hypothalamic area, anterior part
AHC	anterior hypothalamic area, central part
AHiAL	amygdalohippocampal area, anterolateral part
AHiPM	amygdalohippocampal area, posteromedial part
AHP	anterior hypothalamic area, posterior part
APir	amygdalopiriform transition area
Arc	arcuate hypothalamic nucleus
ArcL	arcuate hypothalamic nucleus, lateral part
ArcLP	arcuate hypothalamic nucleus, lateroposterior part
ArcMP	arcuate hypothalamic nucleus, medial posterior part
AStr	amygdalostriatal transition area
BLA	basolateral amygdaloid nucleus, anterior part
BLP	basolateral amygdaloid nucleus, posterior part
BLV	basolateral amygdaloid nucleus, ventral part
BMA	basolateral amygdaloid nucleus, anterior part
BMP	basomedial amygdaloid nucleus, posterior part
CeC	central amygdaloid nucleus, capsular part
CeL	central amygdaloid nucleus, lateral division
CeM	central amygdaloid nucleus, medial division
CeMAD	central amygdaloid nucleus, medial division, anterodorsal part
CeMPV	central amygdaloid nucleus, medial posteroventral part
cp	cerebral peduncle, basal part
CxA	cortex-amygdala transition zone
DM	dorsomedial hypothalamic nucleus
DMC	dorsomedial hypothalamic nucleus, compact part
DMD	dorsomedial hypothalamic nucleus, dorsal part
DMV	dorsomedial hypothalamic nucleus, ventral part
DTM	dorsal tuberomammillary nucleus
I	intercalated nuclei of the amygdala
IM	intercalated amygdaloid nucleus, main part
IPAC	interstitial nucleus of the posterior limb of the anterior commissure
LA	lateroanterior hypothalamic nucleus
LaDL	lateral amygdaloid nucleus, dorsolateral part
LaVL	lateral amygdaloid nucleus, ventrolateral part
LaVM	lateral amygdaloid nucleus, ventromedial part
LH	lateral hypothalamic area
LM	lateral mammillary nucleus
MCLH	magnocellular nucleus of the lateral hypothalamus
MeA	medial amygdaloid nucleus, anterior part
MeAD	medial amygdaloid nucleus, anteriodorsal part
MePD	medial amygdaloid nucleus, posterodorsal part
MePV	medial amygdaloid nucleus, posteroventral part
ML	medial mammillary nucleus, lateral part
MM	medial mammillary nucleus, medial part
MMn	medial mammillary nucleus, median part
MPA	medial preoptic area
mt	mammillothalamic tract
mtg	mammillotegmental tract
MTu	medial tuberal nucleus
ns	nigrostriatal bundle
opt	optic tract
PaAP	paraventricular hypothalamic nucleus, anterior parvicellular part
PaDC	paraventricular hypothalamic nucleus, dorsal cap
PaLM	paraventricular hypothalamic nucleus, lateral magnocellular part
PaMM	paraventricular hypothalamic nucleus, medial magnocellular part
PaMP	paraventricular hypothalamic nucleus, medial parvicellular part
PaPo	paraventricular hypothalamic nucleus, posterior part
PaV	paraventricular hypothalamic nucleus, ventral part
Pe	periventricular hypothalamic nucleus
PeF	perifornical nucleus
PH	posterior hypothalamic area
PLCo	posterolateral cortical amygdaloid nucleus
PMCo	posteromedial cortical amygdaloid nucleus (C3)
PMD	premammillary nucleus, dorsal part
PMV	premammillary nucleus, ventral part
PSTh	parasubthalamic nucleus
RCh	retrochiasmatic area
SCh	suprachiasmatic nucleus
SChDM	suprachiasmatic nucleus, dorsomedial part
SChVL	suprachiasmatic nucleus, ventrolateral part
SHy	septohypothalamic nucleus
SMT	submammillothalamic nucleus
SO	supraoptic nucleus
SOR	supraoptic nucleus, retrochiasmatic part
sox	supraoptic decussation
SPa	subparaventricular zone of the hypothalamus
Stg	stigmoid hypothalamic nucleus
Subl	subincertal nucleus
SuM	supramammillary nucleus
SuML	supramammillary nucleus, lateral part
SuMM	supramammillary nucleus, medial part
sumx	supramammillary decussation
TC	tuber cinereum area
Te	terete hypothalamic nucleus
VMH	ventromedial hypothalamic nucleus
VMHC	ventromedial hypothalamic nucleus, central part
VMHDM	ventromedial hypothalamic nucleus, dorsomedial part
VMHVL	ventromedial hypothalamic nucleus, ventrolateral part
ZI	zona incerta
ZID	zona incerta, dorsal part
ZIV	zona incerta, ventral part
Midbrain and pons	
3 N	oculomotor nucleus
3PC	oculomotor nucleus, parvicellular part
ATg	anterior tegmental nucleus
bic	brachium of the inferior colliculus
BIC	nucleus of the brachium of the inferior colliculus
CGA	central gray, alpha part
CGPn	central gray of the pons
CIC	central nucleus of the inferior colliculus
CnF	cuneiform nucleus
csc	commissure of the superior colliculus
DCIC	dorsal cortex of the inferior colliculus
Dk	nucleus of Darkschewitsch
DLPAG	dorsolateral periaqueductal gray
DMPAG	dorsomedial periaqueductal gray
DMPn	dorsomedial pontine nucleus
DMTg	dorsomedial tegmental area
DpG	deep gray layer of the superior colliculus
DpMe	deep mesencephalic nucleus
DpWh	deep white layer of the superior colliculus
DRC	dorsal raphe nucleus, caudal part
DRI	dorsal raphe nucleus, interfascicular part
DRV	dorsal raphe nucleus, ventral part
DRVL	dorsal raphe nucleus, ventrolateral part
DTgC	dorsal tegmental nucleus, central part
DTgP	dorsal tegmental nucleus, pericentral part
ECIC	external cortex of the inferior colliculus
EMi	epimicrocellular nucleus
EW	Edinger-Westphal nucleus
IF	interfascicular nucleus
InC	interstitial nucleus of Cajal
InCG	interstitial nucleus of Cajal, greater part
InCo	intercollicular nucleus
InG	intermediate gray layer of the superior colliculus
InWh	intermediate white layer of the superior colliculus
IPC	interpeduncular nucleus, caudal subnucleus
IPDL	interpeduncular nucleus, dorsolateral subnucleus
IPDM	interpeduncular nucleus, dorsomedial subnucleus
IPF	interpeduncular fossa
IPI	interpeduncular nucleus, intermediate subnucleus
IPL	interpeduncular nucleus, lateral subnucleus
IPR	interpeduncular nucleus, rostral subnucleus
KF	Ko¨lliker-Fuse nucleus
LC	locus coeruleus
LDTg	laterodorsal tegmental nucleus
LDTgV	laterodorsal tegmental nucleus, ventral part
lfp	longitudinal fasciculus of the pons
LPAG	lateral periaqueductal gray
LPBC	lateral parabrachial nucleus, central part
LPBS	lateral parabrachial nucleus, superior part
LPBV	lateral parabrachial nucleus, ventral part
MA3	medial accessory oculomotor nucleus
MCPC	magnocellular nucleus of the posterior commissure
MiTg	microcellular tegmental nucleus
ml	medial lemniscus
mlf	medial longitudinal fasciculus
MnR	median raphe nucleus
Mo5	motor trigeminal nucleus
mp	mammillary peduncle
MPB	medial parabrachial nucleus
MT	medial terminal nucleus of the accessory optic tract
Op	optic nerve layer of the superior colliculus
Pa4	paratrochlear nucleus
PAG	periaqueductal gray
PBP	parabrachial pigmented nucleus
PCom	nucleus of the posterior commissure
PMnR	paramedian raphe nucleus
Pn	pontine nuclei
PN	paranigral nucleus
PnC	pontine reticular nucleus, caudal part
PnO	pontine reticular nucleus, oral part
PnV	pontine reticular nucleus, ventral part
PPTg	pedunculopontine tegmental nucleus
RC	raphe cap
RLi	rostral linear nucleus of the raphe
RMC	red nucleus, magnocellular part
RPC	red nucleus, parvicellular part
RPF	retroparafascicular nucleus
RPO	rostral periolivary region
RRF	retrorubral field
RtTg	reticulotegmental nucleus of the pons
RtTgP	reticulotegmental nucleus of the pons, pericentral part
Sag	sagulum nucleus
SC	superior colliculus
scp	superior cerebellar peduncle (brachium conjunctivum)
SNC	substantia nigra, compact part
SNL	substantia nigra, lateral part
SNR	substantia nigra, reticular part
Su3	supraoculomotor periaqueductal gray
Su3C	supraoculomotor cap
SubB	subbrachial nucleus
SuG	superficial gray layer of the superior colliculus
ts	tectospinal tract
VLPAG	ventrolateral periaqueductal gray
VLTg	ventrolateral tegmental area
VTA	ventral tegmental area
vtgx	ventral tegmental decussation
VTRZ	visual tegmental relay zone
xscp	decussation of the superior cerebellar peduncle
Zo	zonal layer of the superior colliculus
Medulla	
7 N	facial nucleus
10 N	dorsal motor nucleus of vagus
12 N	hypoglossal nucleus
Amb	ambiguus nucleus
AP	area postrema
CeCv	central cervical nucleus
cu	cuneate fasciculus
Cu	cuneate nucleus
DMSp5	dorsomedial spinal trigeminal nucleus
DPGi	dorsal paragigantocellular nucleus
DPO	dorsal periolivary region
ECu	external cuneate nucleus
EVe	nucleus of origin of efferents of the vestibular nerve
FVe	F cell group of the vestibular complex
Gi	gigantocellular reticular nucleus
GiA	gigantocellular reticular nucleus, alpha part
GiV	gigantocellular reticular nucleus, ventral part
Gr	gracile nucleus
icp	inferior cerebellar peduncle (restiform body)
In	intercalated nucleus of the medulla
InM	intermedius nucleus of the medulla
IOB	inferior olive, subnucleus B of medial nucleus
IOBe	inferior olive, subnucleus B of medial nucleus
IOC	inferior olive, subnucleus C of medial nucleus
IOD	inferior olive, dorsal nucleus
IOK	inferior olive, cap of Kooy of the medial nucleus
IOVL	inferior olive, ventrolateral protrusion
IRt	intermediate reticular nucleus
Li	linear nucleus of the medulla
LPGi	lateral paragigantocellular nucleus
LRt	lateral reticular nucleus
LRtPC	lateral reticular nucleus, parvicellular part
LSO	lateral superior olive
LVe	lateral vestibular nucleus
LVPO	lateroventral periolivary nucleus
MdD	medullary reticular nucleus, dorsal part
MdV	medullary reticular nucleus, ventral part
MVe	medial vestibular nucleus
MVeMC	medial vestibular nucleus, magnocellular part
MVePC	medial vestibular nucleus, parvicellular part
PCRt	parvicellular reticular nucleus
PCRtA	parvicellular reticular nucleus, alpha part
PMn	paramedian reticular nucleus
PPy	parapyramidal nucleus
Pr	prepositus nucleus
Pr5DM	principal sensory trigeminal nucleus, dorsomedial part
Pr5VL	principal sensory trigeminal nucleus, ventrolateral part
PSol	parasolitary nucleus
py	pyramidal tract
pyx	pyramidal decussation
RAmb	retroambiguus nucleus
RMg	raphe magnus nucleus
Ro	nucleus of Roller
ROb	raphe obscurus nucleus
RPa	raphe pallidus nucleus
RVL	rostroventrolateral reticular nucleus
Sol	nucleus of the solitary tract
SolC	nucleus of the solitary tract, commissural part
SolCe	nucleus of the solitary tract, central part
SolDL	solitary nucleus, dorsolateral part
SolDM	nucleus of the solitary tract, dorsomedial part
SolG	nucleus of the solitary tract, gelatinous part
SolI	nucleus of the solitary tract, interstitial part
SolIM	nucleus of the solitary tract, intermediate part
SolM	nucleus of the solitary tract, medial part
SolV	solitary nucleus, ventral part
SolVL	nucleus of the solitary tract, ventrolateral part
sp5	spinal trigeminal tract
Sp5C	spinal trigeminal nucleus, caudal part
Sp5I	spinal trigeminal nucleus, interpolar part
Sp5O	spinal trigeminal nucleus, oral part
Sp5ODM	spinal trigeminal nucleus, oral part, dorsomedial division
Sp5OVL	spinal trigeminal nucleus, oral part, ventrolateral division
SPO	superior paraolivary nucleus
SpVe	spinal vestibular nucleus
SuVe	superior vestibular nucleus
VCA	ventral cochlear nucleus, anterior part
vsc	ventral spinocerebellar tract
X	nucleus X
Cerebellum	
1Cb	1st Cerebellar lobule
2Cb	2nd Cerebellar lobule
3Cb	3rd Cerebellar lobule
4&5Cb	4&5th Cerebellar lobules
6Cb	6th Cerebellar lobule
7Cb	7th Cerebellar lobule
8Cb	8th Cerebellar lobule
9Cb	9th Cerebellar lobule
10Cb	10th Cerebellar lobule
Crus1	crus 1 of the ansiform lobule
Crus2	crus 2 of the ansiform lobule
FI	flocculus
PFI	paraflocculus
PM	paramedian lobule
Sim	simple lobule
Ventricular zones	
3 V	3^rd^ ventricle
OV	olfactory ventricle (olfactory part of lateral ventricle)
OV	olfactory ventricle (olfactory part of lateral ventricle)
SVZ	subventricular zone
